# Etymologia: Rabies

**DOI:** 10.3201/eid1807.ET1807

**Published:** 2012-07

**Keywords:** etymologia, rabies, Lyssavirus, encephalomyelitis, feral dog, soldier

## Rabies [ra′bēz]

From the Latin *rabere* (to rage), which may have roots in the Sanskrit *rabhas* (to do violence). Acute progressive fatal encephalomyelitis caused by neurotropic viruses in the genus Lyssavirus―from the Greek *lyssa* (frenzy or madness). In Greek mythology, Lyssa was the goddess of rage, fury, and rabies, known for driving mad the dogs of the hunter Acteon and causing them to kill their master.

Democritus (460–370) described rabies, and Hippocrates is believed to refer to the disease when he said that “persons in a frenzy drink very little, are disturbed and frightened, tremble at the least noise, or are seized with convulsions.” According to Aristotle, “Dogs suffer from the madness. This causes them to become irritable and all animals they bite to become diseased.” The disease in humans was characterized by hydrophobia, in which the sick person was simultaneously tormented with thirst and fear of water. The Roman writer Cardanus described the saliva from a rabid dog as a *virus*, the Latin word for poison.

Canine rabies has been eliminated in the continental United States. However, dog bites remain a concern for travelers to areas where the disease is enzootic.
